# Design of Protease-Responsive Antifungal Liposomal Formulation Decorated with a Lipid-Modified Chitin-Binding Domain

**DOI:** 10.3390/ijms25073567

**Published:** 2024-03-22

**Authors:** Hendra Saputra, Muhammad Safaat, Pugoh Santoso, Rie Wakabayashi, Masahiro Goto, Toki Taira, Noriho Kamiya

**Affiliations:** 1 Department of Applied Chemistry, Graduate School of Engineering, Kyushu University, 744 Motooka, Fukuoka 819-0395, Japan; saputra.hendra.970@s.kyushu-u.ac.jp (H.S.); safaat.muhammad.304@s.kyushu-u.ac.jp (M.S.); santoso.pugoh.586@m.kyushu-u.ac.jp (P.S.); wakabayashi.rie.122@m.kyushu-u.ac.jp (R.W.); m-goto@mail.cstm.kyushu-u.ac.jp (M.G.); 2 Division of Biotechnology, Center for Future Chemistry, Kyushu University, 744 Motooka, Fukuoka 819-0395, Japan; 3 Department of Bioscience and Biotechnology, Faculty of Agriculture, University of the Ryukyus, Nishihara-cho, Okinawa 903-0213, Japan; tokey@agr.u-ryukyu.ac.jp

**Keywords:** antifungal formulation, AmBisome, *C. albicans*, chitinase, lipid conjugation

## Abstract

*Candida albicans* is a prevalent fungal pathogen that displays antibiotic resistance. The polyene antifungal amphotericin B (AmB) has been the gold standard because of its broad antifungal spectra, and its liposomal formulation, AmBisome, has been used widely and clinically in treating fungal infections. Herein, we explored enhancing the antifungal activity of AmBisome by integrating a small chitin-binding domain (LysM) of chitinase A derived from *Pteris ryukyuensis*. LysM conjugated with a lipid (LysM–lipid) was initially prepared through microbial transglutaminase (MTG)-mediated peptide tag-specific conjugation of LysM with a lipid–peptide substrate. The AmBisome formulation modified with LysM–lipid conjugates had a size distribution that was comparable to the native liposomes but an increased zeta potential, indicating that LysM–lipid conjugates were anchored to AmBisome. LysM–lipid-modified AmBisome exhibited long-term stability at 4 °C while retaining the capacity to bind chitin. Nevertheless, the antifungal efficacy of LysM–lipid-modified AmBisome against *C. albicans* was modest. We then redesigned a new LysM–lipid conjugate by introducing a peptide linker containing a thrombin digestion (TD) site at the C-terminus of LysM (LysM–TD linker–lipid), thereby facilitating the liberation of the LysM domain from AmBisome upon the addition of thrombin. This new AmBisome formulation anchored with LysM–TD linker–lipid exhibited superior performance in suppressing *C. albicans* growth in the presence of thrombin compared with the LysM–lipid formulation. These results provide a platform to design stimuli-responsive AmBisome formulations that respond to external environments and thus advance the treatment of pathogenic fungi infections.

## 1. Introduction

Finding effective, specific, and safe compounds is urgently required to treat various infectious diseases, which the recent coronavirus pandemic has highlighted. Antifungals target fungi and identifying new antifungals with a broad spectrum of fungicidal activity is crucial. Using either chemical or biological approaches, antifungal formulations have been developed by researchers to enhance antifungal activity and reduce toxicity [1,2]. Amphotericin B (AmB) is a recognized antifungal with a broad spectrum of activity against yeast and molds. One mechanism of AmB activity is the disruption of the target cell membrane (i.e., pore formation) due to the interaction between AmB and ergosterol [3,4,5]. More than half a century ago, AmB was used as a reference for treating fungal infections; however, AmB has been increasingly replaced by AmBisome^®^, Abelcet^®^ (Amphotericin B Lipid Complex, ABLC) and Amphocil^®^ (Amphotericin B Colloidal Dispersion, ABCD), which have the same activity profile as AmB but are safer [6]. AmBisome^®^, a lipid-based formulation, provides a single lipid-bilayer liposomal drug delivery system for AmB. Its characteristics include a particle distribution index (PDI) of 0.12, a zeta potential of −53.5 mV (negative charge) and a particle diameter of approximately 100 nm [7,8]. In addition, AmBisome is one of three FDA-approved amphotericin B formulations that are now available on the market [8,9]. This liposomal formulation has been clinically used and can be combined with amphiphilic substances that interact with the bilayer membrane [10].

*Candida albicans* is a prevalent pathogenic fungus that usually colonizes the surface of the skin, cornea, oral cavity, urinary tract, gastrointestinal tract and female urogenital tract [11,12,13,14,15]. HIV-positive individuals, cancer patients and transplant recipients are all susceptible to *C. albicans* infections [16,17,18,19,20]. *C. albicans* is resistant to several antifungal drugs because of its ability to form biofilms [21,22,23,24,25]. This pathogenic fungus was included in the severe-grade group of 19 fungi that pose the greatest threat to public health [26]. This grading was the first time a fungus was included on the list of priority diseases published by the World Health Organization (WHO) in 2022. Therefore, development of new antifungal agents for treating *Candida* infections with high efficacy and low toxicity has been considered. Lipotrophic molecules, such as biomaterials, liposomes and lipid complexes, as well as ionic liquid, were added in the intravenous formulations for the treatment of fungal infections [27,28,29]. Moreover, an alternative formulation of AmB with increased antifungal activity can be obtained by formulating it with a therapeutic protein such as chitinase to target fungal cell walls, which are made of chitin. 

Chitin, a complex polysaccharide composed of β-1,4-N-acetylglucosamine monomers, is an essential component of fungal cell walls and a promising antifungal drug target. Chitinase, which generally consists of a catalytic domain and a chitin-binding domain, can be used to break down chitin into its oligomers and/or monomers, thereby destroying the fungal cell wall structure and ultimately inhibiting cell growth [30]. Furthermore, chitin is also absent in humans, thus making antifungal drugs that target chitin an ideal strategy. Previous research demonstrated how chitinase A from *Pteris ryukyuensis* (PrChiA) [31] may be useful in antifungal formulations. Specifically, a small chitin-binding domain of PrChiA called LysM was essential for the antifungal activity of PrChiA. In addition, cell surface hydrophobicity, an important cellular biophysical parameter that affects both cell–cell and cell–surface interactions, influences the adhesive properties of the opportunistic fungal pathogen *C. albicans*. Hydrophobic proteins present in the *C. albicans* cell wall [32] can affect fungal virulence and biofilm formation [33,34] and are targets of numerous antifungal formulations [35,36]. For this reason, the hydrophobic drug formulation could be more effective in associating the fungal cell wall and cell membrane. Anchoring proteins to cell membranes can be achieved by modifying proteins with lipids (e.g., fatty acids, isoprenoids, sterols and phospholipids) [37]. In particular, increasing the alkyl chain length of the lipid from C12 to C18 had a marked difference in the affinity of the protein–lipid conjugate to the cell membrane [37]. A recent report demonstrated a strategy of conjugating chitinase with a substance that increases the hydrophobicity of the chitinase and showed that artificial palmitoylated LysM suppressed the growth of *Trichoderma viride* [38,39]. 

Herein, we explored the potential of our liposomal formulation containing a combination of AmBisome and LysM–lipid conjugates to inhibit the growth of *C. albicans*. After characterization and validation, we confirmed the design of a new protease-responsive antifungal liposomal formulation decorated with a lipid-modified chitin-binding domain.

## 2. Results

### 2.1. Conjugation of LysM with Different Types of Lipid Substrates

**Figure 1:**
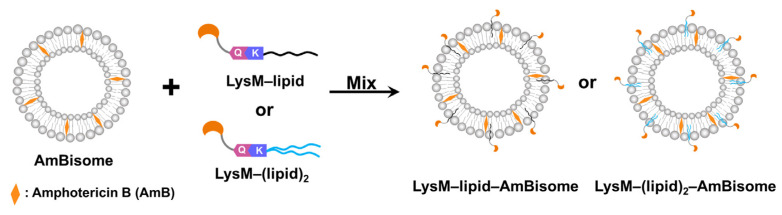
Schematic illustration of the LysM domain with AmBisome. Mixing LysM–lipid or LysM–(lipid)_2_ with AmBisome generates LysM–lipid–AmBisome formulations.

Microbial transglutaminase (MTG) was used to conjugate LysM fused with an MTG-reactive Gln-containing peptide (LysM–Q) to a different type of lipid–peptide substrate. MTG-catalyzed bioconjugation of LysM–Q and mono-lipidized (C12–K, C14–K and C16–K) lipid substrates as well as LysM–Q and di-lipidized ((C12)_2_–K, (C14)_2_–K) lipid substrates (Lipid-Ks) was conducted to obtain LysM–C12, LysM–C14, LysM–C16, LysM–(C12)_2_, LysM–(C14)_2_ and LysM–(C16)_2_ “https://biorxiv.org/cgi/content/short/2024.03.04.583322v1 (accessed on 4 March 2024)”. All the LysM–lipid conjugates were mixed with AmBisome to obtain LysM–lipid–AmBisome (Figure 1).

### 2.2. Dynamic Light Scattering (DLS) Analysis of LysM–Lipid–AmBisome

**Figure 2:**
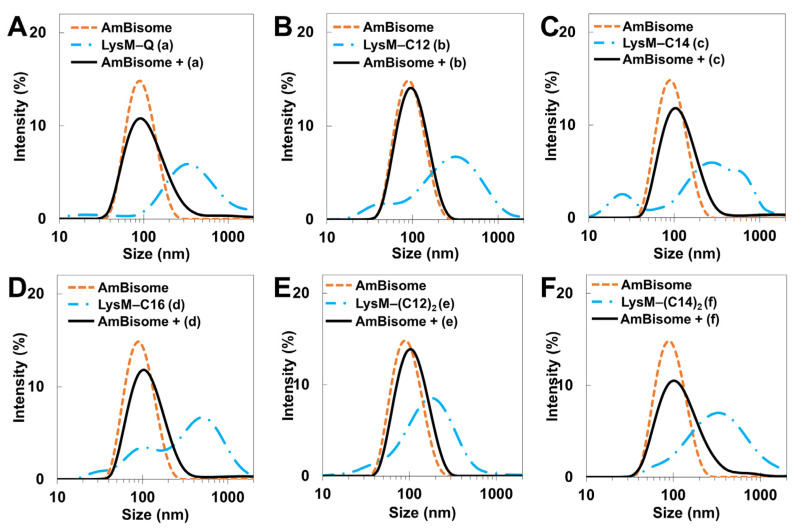
DLS measurements: (**A**) LysM–Q; (**B**) LysM–C12; (**C**) LysM–C14; (**D**) LysM–C16; (**E**) LysM–(C12)_2_; and (**F**) LysM–(C14)_2_. The letters (a), (b), (c), (d), (e), and (f) represent LysM–Q, LysM–C12, LysM–C14, LysM–C16, LysM–(C12)_2_, and LysM–(C14)_2_, respectively.

The size distribution of LysM–lipid–AmBisomes was examined using DLS to obtain information on the status of LysM–lipids in an aqueous solution with AmBisome (Figure 2). Both LysM–Q and LysM–lipids were likely to form aggregates in the solution; however, a single major peak was observed in all cases with AmBisome, suggesting an association of LysM–Q and LysM–lipid conjugates with AmBisome. The size of the liposomes increased slightly when LysM–lipid or LysM–(lipid)_2_ was combined with AmBisome. Furthermore, the negative charge of the zeta potential decreased (Figure S1), indicating the integration of LysM–lipid conjugates on the surface of AmBisome. Changes in liposomal size and the zeta potential represent an extension of the LysM–C16 study that was reported previously [39]. 

**Figure 3:**
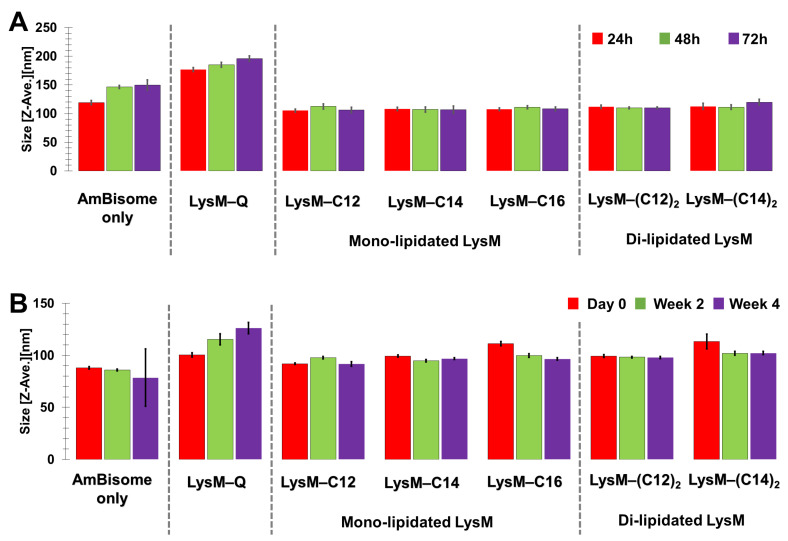
Stability of the mono-lipidated LysM or di-lipidated LysM with AmBisome formulations after (**A**) three days of incubation at 25 °C and (**B**) four weeks of storage at 4 °C.

The stability of the LysM–lipid–AmBisome preparations was examined at 25 °C for 3 days, with a size analysis conducted every 24 h (Figure 3A and Figure S2). In general, AmBisome had a homogeneous size distribution when combined with LysM–lipids. Moreover, storage under controlled settings showed that the LysM–lipid–AmBisome preparations were stable at 4 °C for four weeks, with a size analysis conducted every two weeks (Figure 3B). Compared to LysM–lipid, the LysM–Q sample aggregated more readily under the storage conditions, possibly because of nonspecific interactions with AmBisome due to the lack of lipids.

### 2.3. Confocal Laser Scanning Microscopy (CLSM) Analysis

**Figure 4:**
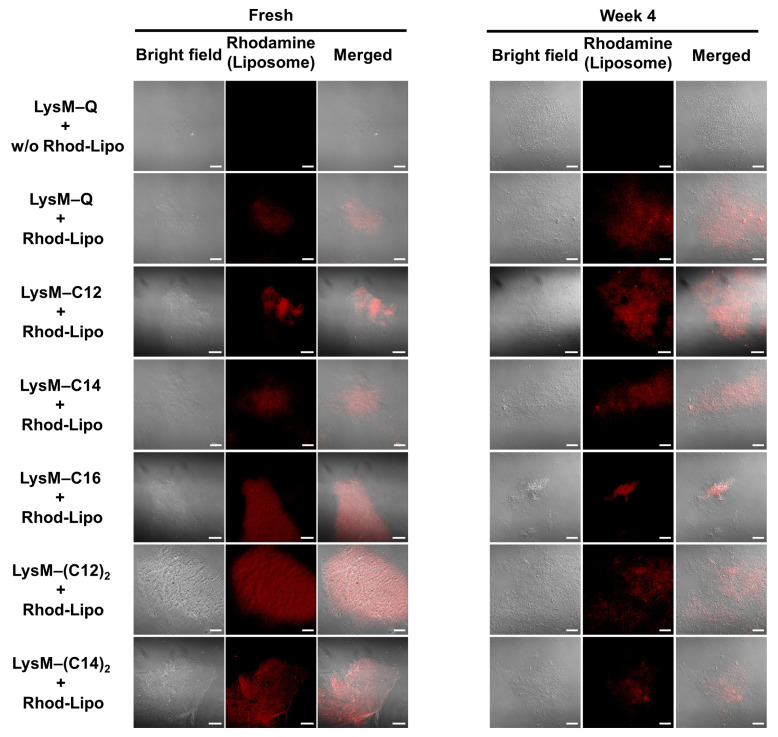
CLSM analysis of rhodamine-labeled liposomes (Rhod-Lipo) (total lipid concentration of 50 μM) with LysM–Q (1 μM) or LysM–lipid (1 μM) in the presence of 0.5% α-chitin in 20 mM sodium phosphate buffer, pH 7.4, at 25 °C (bars: 20 μm). Red color indicates the red fluorescence from Rhod-lipo adsorbed on α-chitin.

The chitin-binding ability of LysM–lipids on AmBisome was evaluated. A liposome labeled with a rhodamine-modified lipid (Rhod-Lipo) was prepared, and LysM–Q or LysM–lipid was anchored to this liposome. This experiment enabled the visualization of the binding of LysM to chitin. The CLSM results showed that LysM on AmBisome bound to chitin in either the LysM–Q or LysM–lipid forms (Figure 4). Rhod-Lipo showed little binding to chitin (Figure S3), and LysM–lipid-modified Rhod-Lipo showed higher fluorescence intensity than the mixture of LysM–Q and Rhod-Lipo (Figure S4), implying the oriented anchoring of LysM–lipids on the liposome. The formulations maintained their chitin-binding capacity after four weeks of storage at 4 °C.

### 2.4. Evaluation of the Antifungal Activity of LysM–Lipid–AmBisome against C. albicans

**Figure 5:**
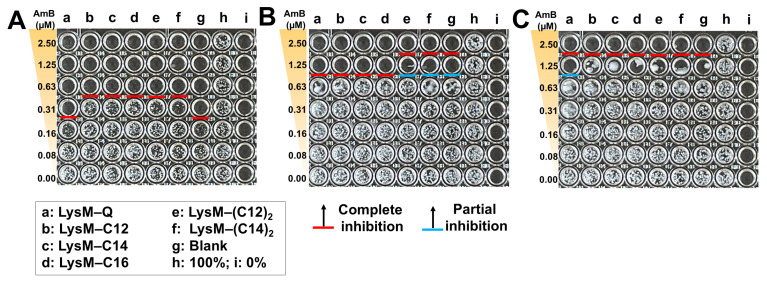
Antifungal activity of AmBisome with LysM–Q or LysM–lipid (1 μM) against *C. albicans* (10,000 cells/mL) after treatment in 20 mM sodium phosphate buffer, pH 7.4, at 35 °C and 90% humidity. Images of the 96-well plates after culturing *C. albicans* in the presence of 0–2.5 μM AmB, with 1 μM of each sample: (**A**) 24 h; (**B**) 48 h; and (**C**) 72 h.

Suppression of *C. albicans* growth by the LysM–AmBisome preparations was examined. AmBisome and chitinase preparations were loaded into wells with different doses of encapsulated AmB (0–2.5 μM), a set of concentrations of LysM–Q or LysM–lipid (1 μM) and 10,000 cells/mL of *C. albicans* (Figure 5). The growth of *C. albicans* was inhibited within 24 h when the AmB concentration was 0.31 μM in the AmBisome or AmBisome formulation with LysM–Q. Cell growth was inhibited for all treatments with LysM–lipid and AmBisome at 0.63 μM AmB. This is near the typical minimum inhibitory concentration (MIC) of AmB, which is approximately 1 μg/mL for a wide range of yeast species [40]. Moreover, cell growth was inhibited for all treatments, including AmBisome as the control, as well as Lipid-Ks (Figure S5), at 1.25 μM AmB after 48 h. At 72 h, LysM–Q produced partial inhibition, which was a better outcome than that of other treatments at 1.25 μM AmB. 

### 2.5. Design of LysM–TD Linker–Lipid and DLS Analysis with AmBisome

**Figure 6:**
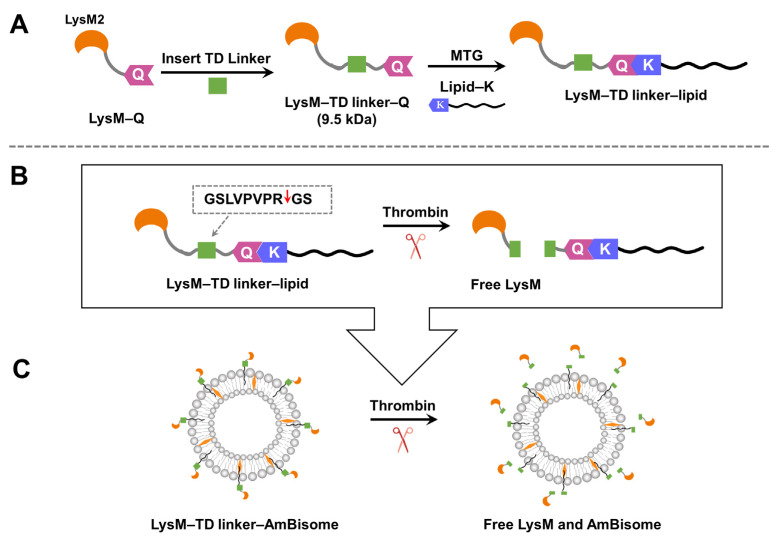
(**A**) The design of the LysM domain with a thrombin-digestive (TD) linker. (**B**) The thrombin linker is cleaved by thrombin. Red arrow indicates the cleavage site. (**C**) Schematic illustrating of the new LysM formulation with the thrombin linker inserted into the AmBisome bilayer and the subsequent release of LysM following cleavage with thrombin.

On the basis of the preceding results, we hypothesized that free LysM–Q had the greatest inhibitory effect against the growth of *C. albicans* when combined with AmBisome. Thus, we designed LysM–Q with a thrombin-digestive (TD) linker to add responsiveness to external environments (LysM–TD linker–Q, Figure 6). The thrombin linker enables the formation of free LysM in the liposomal formulation of AmB within a specific time frame using the proteolytic enzyme thrombin. We prepared a set of LysM–TD linker–lipids with different lipid moieties (Figure S6).

**Figure 7:**
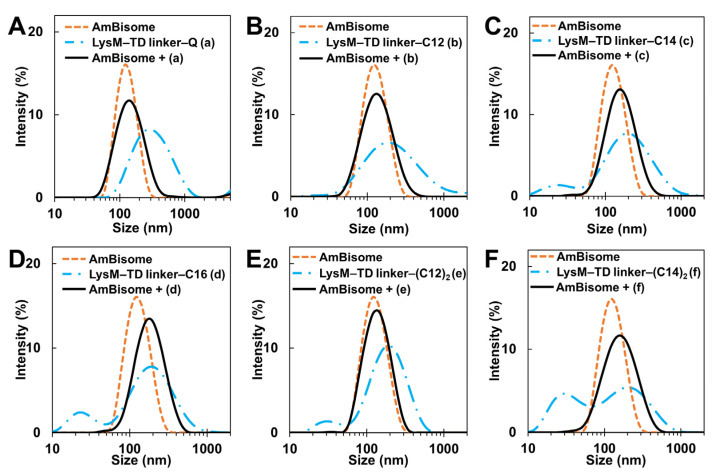
DLS measurements: (**A**) LysM–TD linker–Q; (**B**) LysM–TD linker–C12; (**C**) LysM–TD linker–C14; (**D**) LysM–TD linker–C16; (**E**) LysM–TD linker–(C12)_2_; and (**F**) LysM–TD linker–(C14)_2_. The letters (a), (b), (c), (d), (e), and (f) represent LysM–TD linker–Q, LysM–TD linker–C12, LysM–TD linker–C14, LysM– TD linker–C16, LysM–TD linker–(C12)_2_, and LysM–TD linker–(C14)_2_, respectively.

The size distribution of the LysM–TD linker–lipid-modified AmBisome (LysM–TD linker–AmBisome) was studied using DLS to evaluate the interaction between the LysM–TD linker–lipids and AmBisome in an aqueous solution. As observed for the LysM–lipid–AmBisome preparations (Figure 2), similar size distributions and zeta potentials were observed when examining the LysM–linker–lipid conjugates and AmBisome preparations (Figure S7 and Figure 7).

### 2.6. Antifungal Activity of AmBisome with a New LysM Mutant

**Figure 8:**
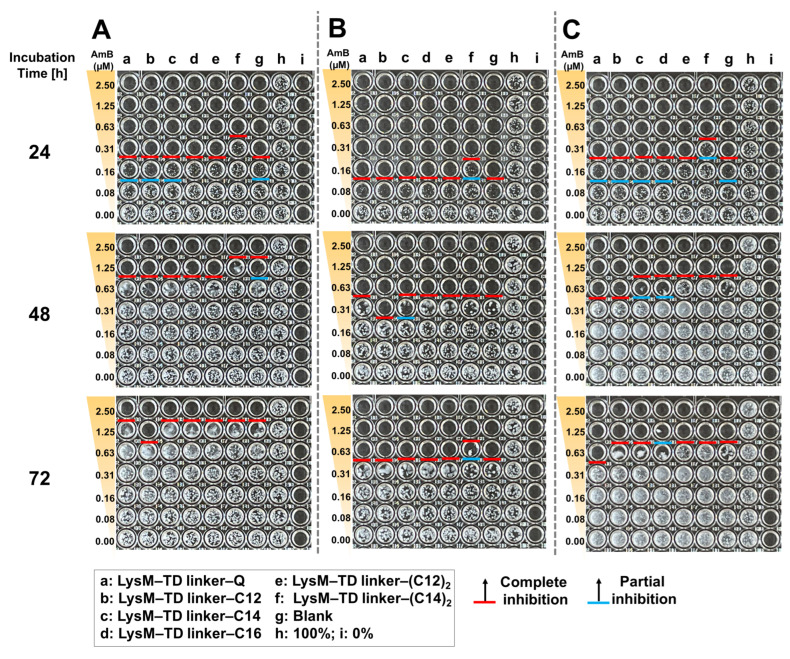
Antifungal activity of AmBisome with LysM–TD linker–Q or LysM–TD linker–lipid (1 μM) and the addition of thrombin (0.1 unit) against *C. albicans* (10,000 cells/mL) after treatment for 24, 48 and 72 h in 20 mM sodium phosphate, pH 7.4, at 35 °C and 90% humidity. *C. albicans* was cultured in 96-well plates in the presence of 0–2.5 μM AmB with 1 μM of each sample. (**A**) Without added thrombin, (**B**) with added thrombin after 0 h incubation and (**C**) with added thrombin after 30 h.

We validated the antifungal activity of a new LysM–TD linker–AmBisome. After a 24 h incubation (Figure 8), the treatments without thrombin that led to complete growth inhibition at an AmB concentration of 0.31 μM were LysM–TD linker–Q, LysM–TD linker–C12 and LysM–TD linker–C14, and this was better than the other treatments that required 0.63 μM AmB. Furthermore, administering thrombin (0.1 unit) at 0 h achieved complete growth inhibition at 0.16 μM AmB for LysM–TD linker–Q, LysM–TD linker–lipid and LysM–TD linker–(lipid)_2_. Only LysM–TD linker–(C14)_2_ led to weaker activity. Partial growth inhibition was also observed at 0.08 μM AmB. Incubation for 48 h and administration of thrombin (0.1 unit) at 0 h revealed that only 0.31 μM AmB was required for LysM–TD linker–C12 to inhibit cell growth. Other samples required 0.63 μM AmB. The administration of thrombin (0.1 unit) at 30 h required 0.63 μM AmB for cell growth inhibition with LysM–TD linker–Q and LysM–TD linker–C12. Partial cell growth inhibition was observed for LysM–TD linker–C14 and LysM–TD linker–C16 at that dosage and also with the addition of thrombin after 30 h. At 72 h, complete growth inhibition at 1.25 μM AmB was observed for LysM–TD linker–C12 without thrombin treatment, whereas with thrombin treatment at 0 h, the AmB concentration required was 0.63 μM, except for LysM–TD linker–(C14)_2_, which required 1.25 μM AmB. It should be noted that the addition of thrombin (0.5 unit) resulted in no antifungal activity (Figure S8). In addition, we confirmed that thrombin cleaved the TD linker independent of time (0 or 30 h) in the absence of AmBisome (Figure S9).

## 3. Discussion

In this report, we investigated changes in the size and surface charge of AmBisome mixed with LysM–C12, LysM–C14, LysM–(C12)_2_ and LysM–(C14)_2_ to focus on the effect of saturated lipids with different cell membrane affinities [37] and compared these results with those obtained previously for LysM–C16 [39]. Furthermore, we monitored the stability of the different formulations during a four-week storage period. The DLS results showed that the liposomes increased in size slightly when the LysM–lipid conjugate was combined with AmBisome (Figure 2), but the size distribution of the formulation was uniform. The formulations were stable because the particle sizes of the LysM–lipid and AmBisome combinations were essentially unchanged after four weeks of storage at 4 °C (Figure 3). Additionally, the negative charge of the ζ-potential decreased following the mixing process for all samples (Figure S1). This resulted because of the lipid bilayer-stabilizing ionic interactions of the mixture’s components [9]. Because the surface charge of *C. albicans* gave a zeta potential of approximately –20 to –35 mV [41], decoration of AmBisome with LysM–lipid conjugates should facilitate its interaction with the fungal cell wall. We also confirmed that LysM–lipid conjugates were incorporated into AmBisome using a method from our previous research [39]. Briefly, a fusion protein consisting of LysM and a green fluorescent protein variant with an MTG-reactive Gln-donor peptide tag (LysM–muGFP–Q) was used as a model protein to estimate the ability of a LysM–lipid to anchor to AmBisome on the basis of a change in fluorescence in the filtrate before and after centrifugation through a membrane filter with a 100 kDa cutoff. The results, shown in Table S1, revealed that LysM–lipid conjugates showed a higher level of anchoring to AmBisome compared to LysM–muGFP–Q, and the anchoring ability of LysM–muGFP–lipid conjugates was enhanced by increasing the length and the number of lipid moieties.

A model system for investigating the localization of the liposomal formulation of AmBisome combined with LysM–C16 on fungi has been demonstrated previously [39]. Here, we used this model system, i.e., a rhodamine-labeled liposomal formulation, to evaluate the chitin-binding activity (Figure 4) of the different liposomal formulations. Red fluorescence around chitin was observed with each lipid tested. Thus, we concluded that modifying the liposome with a LysM–lipid conjugate can modulate binding to chitin.

The antifungal activity of the Q-tagged chitinase domains and the LysM–lipid conjugate with AmBisome was examined (Figure 5). The LysM–lipid or LysM–(lipid)_2_ conjugates, when combined with AmBiosme, led to a lower antifungal activity against *C. albicans* compared to unmodified LysM–Q and AmBisome. This observation indicated that the reduced antifungal activity of lipid-modified LysM may be caused by a reduced ability of LysM to bind to chitin in the cell wall of *C. albicans*. Nonetheless, LysM–lipids in this formulation served as an amphiphilic anchor to guarantee the stability of the system. Low antifungal activity may be the result of the formulation’s high permeability and retention impact [42]. To understand how LysM and AmBisome inhibit fungal growth and optimize the function of lipids in this formulation, we constructed a novel antifungal agent by inserting a thrombin linker in the lipidated LysM construct (Figure 6). Digestion of this construct with thrombin took ~16 h. Using a protease-responsive approach, LysM was released from the lipid, which enabled this LysM domain to readily bind chitin in the cell wall of fungi, and a synergistic effect with AmBisome promoted fungal cell death. The results in Figure 7 showed that the LysM–TD linker–AmBisome particle size was similar to that of the LysM–linker–AmBisome (see Figure 2). Thus, adding a thrombin linker did not affect the size of the formulation. Moreover, the zeta potential of the two AmBisome formulations had the same pattern (Figures S1 and S7). These results indicate that these formulations have similar stability and physical features. 

The antifungal activity of the LysM domain inserted with a thrombin linker was retained (Figure 8). Adding thrombin (0.1 unit) improved the antifungal treatment at 48 h compared to scenarios in which thrombin was not added. This result was particularly apparent when thrombin was added at the start of the experiment (0 h). The administration of additional thrombin at 30 h yielded different results. A possible reason for the difference is that there is a larger number of cells at 30 h of incubation than at 0 h (Figure S9). Enhancing the thrombin amount in the antifungal testing procedure yielded greater results, with LysM–TD linker–C14 and LysM–TD linker–C16 exhibiting partial cell growth inhibition at 0.63 μM AmB. This observation indicates that more LysM was released from AmBisome and that modulating the concentration and timing of thrombin addition can control *C. albicans* growth. Overall, the antifungal activity of the LysM–TD linker–lipid conjugates was higher than that observed for the LysM–lipid conjugates. Furthermore, LysM–TD linker–C12 yielded the best results compared to all treatments at 72 h, with full inhibition of cell growth observed. Thus, LysM enhanced the antifungal activity of AmBisome against *C. albicans*. Additionally, these findings suggest that adding external environment responsiveness to the system improves activity.

## 4. Materials and Methods

### 4.1. Materials

AmBisome was purchased from Sumitomo Pharma Co., Ltd. (Osaka, Japan). AmBisome was reconstituted according to the manufacturer’s instructions. Twelve milliliters H_2_O was added to AmBisome (50 mg), and the sample was shaken. Dissolution was complete when the sample turned yellow and transparent. n-Dodecyl-β-D-maltoside (DDM) and Cell Counting Kit-8 (CCK-8) were purchased from Dojindo (Kumamoto, Japan). Dulbecco’s Modified Eagle medium (D-MEM), antibiotic–antimycotic solution, fetal bovine serum (FBS) and rhodamine B 1,2-dihexadecanoyl-sn-glycero-3-phosphoethanolamine triethylammonium salt (rhodamine-DHPE) were purchased from ThermoFisher Scientific (Waltham, MA USA). Isopropyl β-D-1-thiogalactopyranoside (IPTG) was purchased from Takara Bio, Inc. (Shiga, Japan). Luria-Bertani (LB) broth medium, ammonium peroxodisulfate, 30% acrylamide/bis mixed solution (29:1), tris (hydroxymethyl)aminomethane, tryptone, dried yeast extract, dipotassium hydrogen phosphate and hydrochloric acid were purchased from Nacalai Tesque, Inc. (Kyoto, Japan). Sodium dodecyl sulfate (SDS), glycerol, potassium dihydrogen phosphate and sodium chloride were purchased from Wako Pure Chemical Industries, Ltd. (Osaka, Japan). *N*,*N*,*N*′,*N*′-Tetramethylethylenediamine, HisTrap FF crude 5 mL column, HiTrap Q HP 5 mL column, PD SpinTrap G-25 and Ni Sepharose 6 Fast Flow were purchased from Cytiva (Tokyo, Japan). Thrombin, Amicon Ultra-0.5 (PLBC Ultracel-3 membrane, 3 kDa), Amicon Ultra-0.5 (PLBC Ultracel-3 membrane, 1 kDa) and Amicon^®^ Ultra-15 centrifugal filters (3 kDa MWCO) were from Millipore (Tokyo, Japan). Imidazole was from Sigma-Aldrich (Tokyo, Japan). An α-chitin nanofiber (α-CNF) provided by Professor Shinsuke Ifuku (Tottori University) was used for the chitin-binding assay. The width and degree of deacetylation of α-CNF prepared from crab shell powder were 10–20 nm and 3.9%, respectively [43]. *C. albicans* (Robin) Berkhout (MYA-2876) was purchased from Summit Pharmaceuticals International Corporation (Tokyo, Japan) through Bioresource Materials Distribution Service.

### 4.2. Expression and Purification of Recombinant Proteins

MTG, LysM–Q, LysM–TD linker–Q and LysM–muGFP–Q proteins were prepared and used following the same methods reported previously [38,39].

### 4.3. Fmoc Solid Phase Peptide Synthesis

The lipid–peptide substrates, lipid–G3S–RHK–NH_2_ and (lipid)_2_–KG3S–RHK–NH_2_, were produced manually using an established Fmoc solid phase peptide synthesis method in accordance with a previous study [37].

### 4.4. MTG-Catalyzed Lipid Conjugation Reaction

The bioconjugation of LysM–Q with mono–lipidized (C12–K, C14–K and C16–K) and di–lipidized [(C12)_2_–K, (C14)_2_–K and (C16)_2_–K] lipids were performed in a total reaction volume of 500 µL. The reaction components (100 µM lipid, 10 µM LysM–Q, 1% DDM and 0.1 U/mL MTG) were dissolved in 10 mM Tris–HCl buffer (pH 7.4) and reacted in an incubator shaker. The reaction was performed at 180 rpm and 37 °C for 1 h. Purification was carried out using Ni-Sepharose 6 Fast Flow resin and then a PD SpinTrap G25 column to obtain the conjugated protein. The sample was concentrated.

### 4.5. Particle Size Analysis Using DLS and Zeta Potential

DLS measurements were carried out to determine the particle size and zeta potential of combinations consisting of LysM–lipid conjugates and AmBisome, which was dissolved in 20 mM sodium phosphate buffer (pH 7.4) with a total volume of 100 μL. The experimental approach has been described previously [39].

### 4.6. Chitinase–AmBisome Formulation Antifungal Activity Testing

AmBisome (AmB (0–2.5 μM)), protein, lipid (1 μM) and *C. albicans* (10,000 cells/mL) were mixed in 60 μL Yeast Malt Broth (YMB) in 96-well plates at 35 °C and 90% humidity. *C. albicans* growth was examined after 24, 48 and 72 h. 

### 4.7. Estimation of the Number of LysM–Lipid or LysM–(Lipid)_2_ Conjugates on Lipid Membrane of AmBisome

A mixture of 5 μM LysM–muGFP–Q or LysM–muGFP–lipid with a liposome of AmBisome (5 μM AmB) was combined and then subjected to centrifugation using an Amicon 100 kDa filter at 100,000× *g* for 2 min. Absorbance was measured at 488 nm.

### 4.8. Confocal Laser Scanning Microscopy (CLSM) Analysis

CLSM was performed according to a previous report [39]. In brief, 0.5% α-chitin in 20 mM sodium phosphate buffer, pH 7.4, was mixed with 50 μL of each sample solution and incubated at 25 °C for 1 h. Each sample contained Rhod-Lipo (total lipid concentration of 50 μM) and 1 μM of each protein sample. Subsequently, the sample was incubated at 25 °C for 1 h. The samples underwent a triple washing procedure using a solution of 20 mM sodium phosphate buffer (pH 7.4) before examination using CLSM (LSM700; Carl Zeiss, Oberkochen, Germany).

### 4.9. Antifungal Activity Testing for New LysM with Thrombin

Briefly, the measurement was the same as the previous antifungal activity test method, but an additional thrombin treatment step was included. Treatment with 6 µL thrombin (0.1 unit) was given at 0 h and 30 h after incubation, and *C. albicans* growth was monitored at 24, 48 and 72 h. 

## 5. Conclusions

According to estimates, fungal diseases claim the lives of up to 1.5 million people per year. Moreover, the number of available antifungal drugs is limited, and the prevalence of drug resistance is increasing. The results obtained in this study showed that the formulation of LysM–lipid conjugates with AmBisome have the ability to maintain stability at 25 °C (for antifungal activity) and at 4 °C (for long-term storage). In addition, the introduction of a thrombin digestion site at the C-terminus of LysM could overcome a problem associated with the combined use of AmBisome and LysM-based lipid formulations. It has been shown that the introduction of external stimuli responsiveness could be a choice for effective treatment strategies for fungal diseases.

## Data Availability

Relevant data are contained within the article. Additional data are available from the corresponding author.

## Supplementary Materials

The following supporting information can be downloaded at https://www.mdpi.com/article/10.3390/ijms25073567/s1.
