# Supplementary material for: Design of Protease-Responsive Antifungal Liposomal Formulation Decorated with a Lipid-Modified Chitin-Binding Domain
Source: Int J Mol Sci. 2024 Mar 22;25(7):3567. doi: 10.3390/ijms25073567 (PMC11011847; doi:10.3390/ijms25073567)
Supplement: Supplementary file 1 [file ijms-25-03567-s001.zip › ijms-2885664-supplementary.pdf]

Supplementary Materials

Table of contents

1	Supplementary information		S2
	1-1.	Amino acid sequence of LysM–Q	S2
	1-2.	Amino acid sequence of LysM–muGFP–Q	S2
	1-3.	Amino acid sequence of LysM–TD linker–Q	S3
2	Supplementary results		S3
	2-1.	Zeta potential LysM–Q and LysM–lipid	S3
	2-2.	Stability of LysM–lipid–AmBisome for three days at 25°C	S3
	2-3.	CLSM Analysis of Rhod-Lipo with α-chitin	S4
	2-4.	Quantitative results of fluorescent intensity of LysM–lipid with Rhod-Lipo	S4
	2-5.	Qualitative results of antifungal activity test for lipid–K	S5
	2-6.	Estimation of anchoring ratio of LysM–muGFP–lipid to AmBisome	S5
	2-7.	Conjugation of Q-Tagged LysM with thrombin linker by MTG	S6
	2-8.	Zeta potential LysM–TD linker–Q and LysM–TD linker–lipid	S6
	2-9.	Qualitative results of intensity of thrombin	S7
	2-10.	Evaluation of the cleavage of TD–linker by thrombin	S7

1. Supplementary information

1-1. Amino acid sequence of LysM–Q (pI/Mw: 6.99 / 8739.65)

MCTTYTIKSGDTCYAISQARGISLSDFESWNAGIDCNNLQIGQVVCVSKPSTSTTPSPTPSSSSNGFYPLQ
MRGGHHHHHH

1-2. Amino acid sequence of LysM–muGFP–Q (pI/Mw: 6.04 / 35078.23)

MCTTYTIKSGDTCYAISQARGISLSDFESWNAGIDCNNLQIGQVVCVSKPSTSTTPSPTPSSSSNGHHHHHH
SKGEELFTGVVPILVELDGDVNGHKFSVRGEGEGDATNGKLTLKFICTTGKLPVPWPTLVTTLTYGVLCFSR
YPDHMKRHDFFKSAMPEGYVQERTISFKDDGTYKTRAEVKFEGDTLVNRIELKGIDFKEDGNILGHKLEYNF
NSHNVYITADKQKNGIKAYFKIRHNVEDGSVQLADHYQQNTPIGDGPVLLPDNHYLSTQSVLSKDPNEKRDH
MVLLEDVTAAGITHGMDELYRGGGGSLLQG

1-3. Amino acid sequence of LysM–TD linker–Q (pI/Mw: 6.04 / 35078.23)

MCTTYTIKSGDTCYAISQARGISLSDFESWNAGIDCNNLQIGQVVCVSKPSTSTTPGSLVPRGSSPTPSSSS
NGFYPLQMRGGHHHHHH

Brown: LysM2 domain
Blue: Linker sequences derived from PrChiA
Purple: Microbial transglutaminase-reactive Gln-containing tag (FQ-tag)
Green: Hexahistidine tag
Dark Green: muGFP
Orange: Microbial transglutaminase-reactive Gln-containing tag (LQ-tag)
Pink: Thrombin linker

2. Supplementary results

2-1. Zeta potential LysM–Q or LysM–lipid

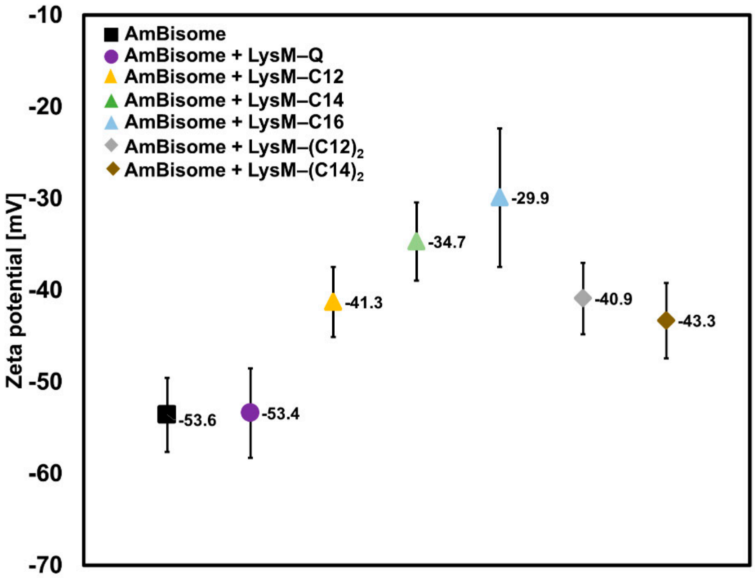

Figure S1. ζ-potential of AmBisome with LysM–Q or LysM–lipid.

2-2. Stability of LysM–lipid–AmBisome for three days at 25°C

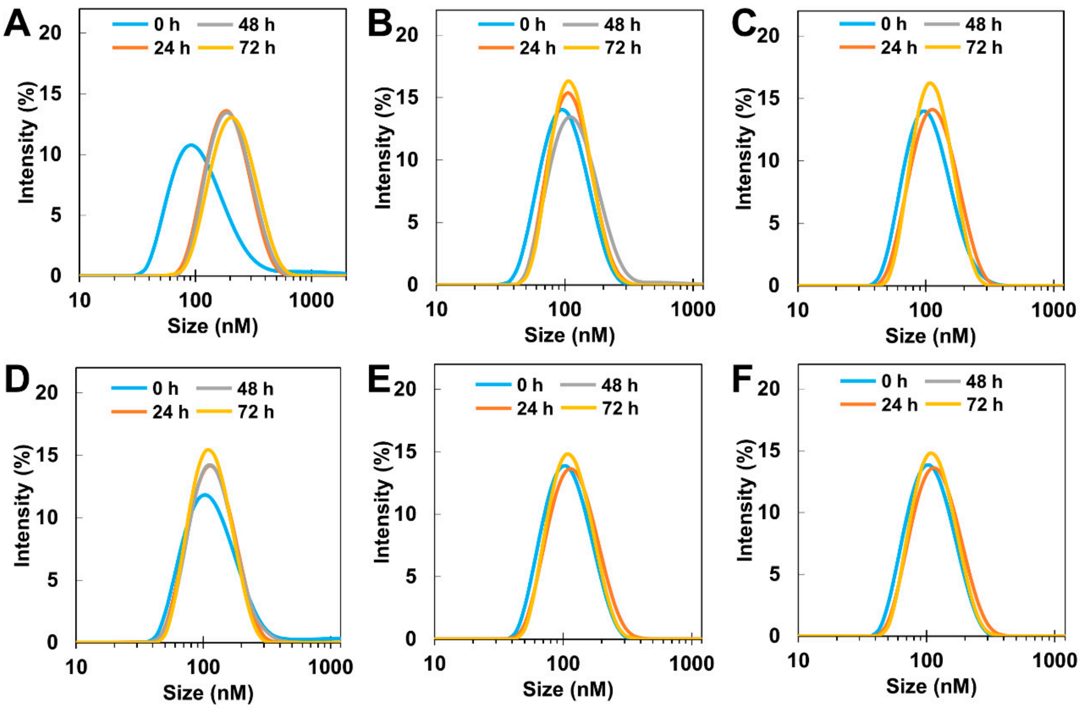

Figure S2. DLS measurements of AmBisome with (**A**) LysM–Q; (**B**) LysM–C12; (**C**) LysM–C14; (**D**) LysM–C16; (**E**) LysM–(C12)$_2$; and (**F**) LysM–(C14)$_2$ during three days of incubation at 25 °C.

2-3. CLSM Analysis of Rhod-Lipo with α-chitin

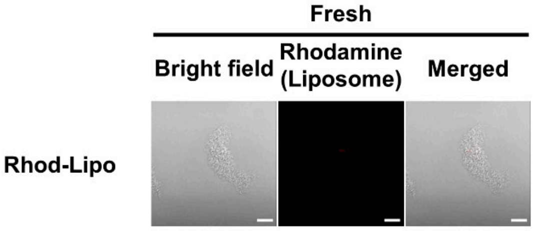

Figure S3. CLSM analysis of Rhod-Lipo (50 μM) in the presence of 0.5% α-chitin in 20 mM sodium phosphate buffer, pH 7.4, at 25 °C (bars: 20 μm).

2-4. Quantitative results of fluorescent intensity of LysM–lipid with Rhod-Lipo

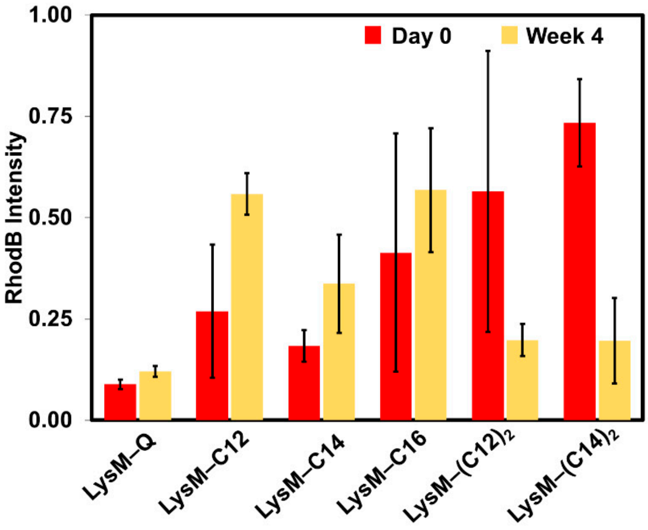

Figure S4. Results of intensity of LysM–lipid labeled with Rhod-Lipo.

2-5. Qualitative results of antifungal activity test for lipid–K

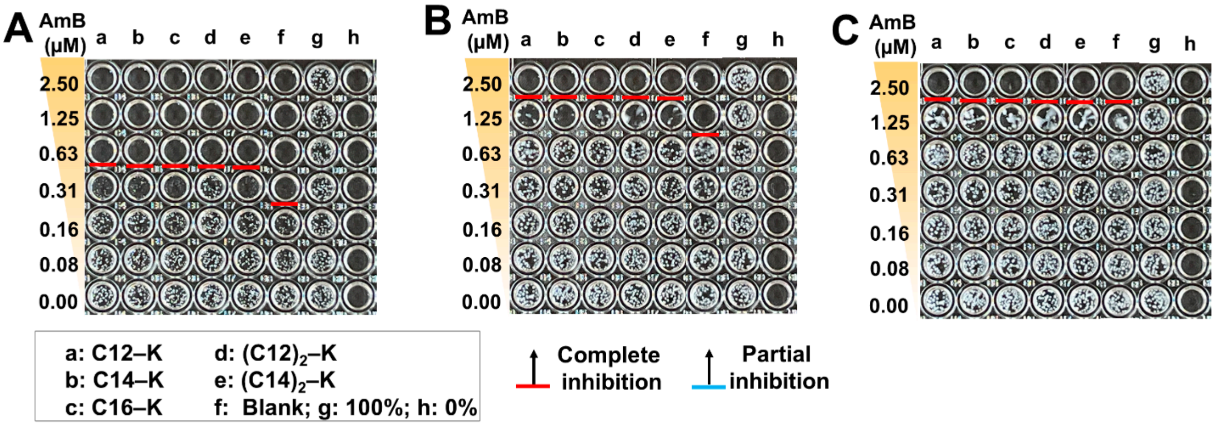

Figure S5. Representative image of a 96-well plate after culturing *C.albicans* in the presence of 0–2.5 μM of AMB with 1 μM of each sample at (A) 24 h, (B) 48 h and (C) 72 h in 20 mM NaPi, pH 7.4, humidity 90% at 35°C.

2-6. Estimation of anchoring ratio of LysM-muGFP-lipid to AmBisome

Table S1. Estimation of the number of LysM–muGFP–lipid or –(lipid)$_2$ on AmBisome

Samples	Anchoring ratio of LysM–Lipid to AmBisome			Estimation of Number of LysM–lipid on AmBisome
	$\text{Total}_{\text{LysM–Lipid}}$	$\text{Anch}_{\text{LysM–Lipid}}$	Anchoring ratio	
**LysM–muGFP–Q**	21.3	1.19	0.0559	457 LysM–muGFP–Q/AmBisome
**LysM–muGFP–C12**	22.5	2.24	0.0996	814 LysM–muGFP–C12/AmBisome
**LysM–muGFP–C14**	21.9	3.57	0.163	1332 LysM–muGFP–C14/AmBisome
**LysM–muGFP–C16**	18.9	3.5	0.1852	1514 LysM–muGFP–C16/AmBisome
**LysM–muGFP–(C12)$_2$**	21.9	6.58	0.3005	2456 LysM–muGFP–(C12)$_2$/AmBisome
**LysM–muGFP–(C14)$_2$**	21.9	6.8	0.3105	2538 LysM–muGFP–(C14)$_2$/AmBisome

Footnote: $\text{Total}_{\text{LysM–lipid}}$, the total amount of LysM–muGFP–lipid used in this experiment; $\text{Anch}_{\text{LysM–lipid}}$, the amount of anchored LysM–muGFP–lipid.

2-7. Conjugation of Q-Tagged LysM with thrombin linker by MTG

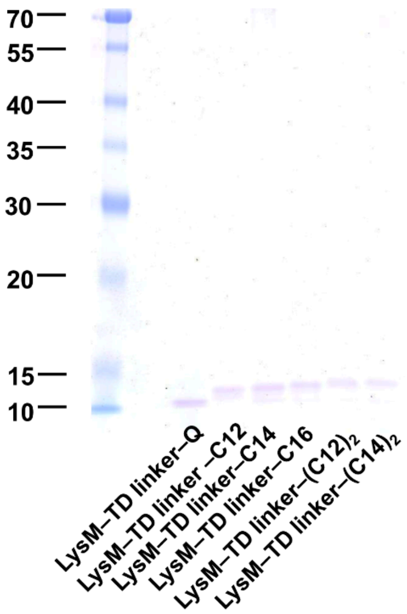

Figure S6. Bioconjugate of the Q-tagged LysMs. (SDS-PAGE analysis results of unmodified LysM–TD linker–Q and LysM–TD linker–Q modified with C12–K, C14–K, C16–K, $(C12)_2$–K, and $(C14)_2$–K by MTG. All conjugation reactions were carried out under conditions of 10 μM LysM–TD linker–Q, 1% DDM, 10 μM Lipid–K, and 0.1 U/mL MTG in 10 mM Tris-HCl (pH 8.0) at 37 °C for 1 h.

2-8. Zeta potential LysM–TD linker–Q or LysM–TD linker–lipid

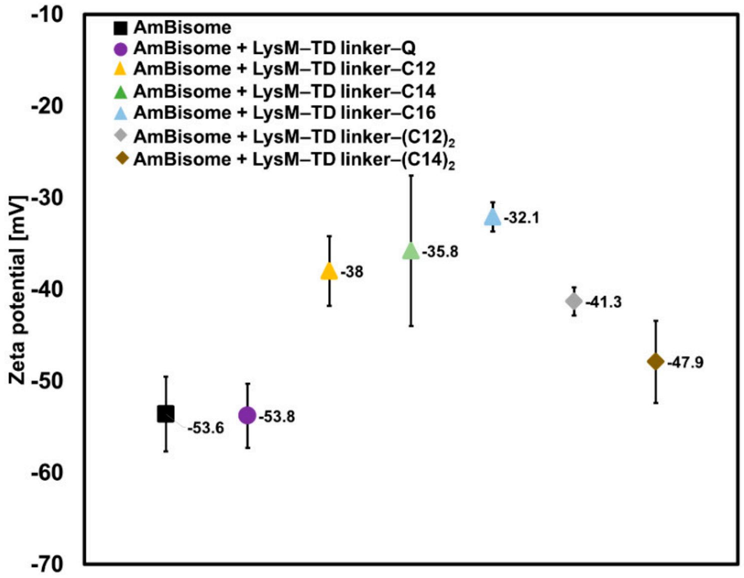

Figure S7. ζ-potential of AmBisome with LysM–TD linker–Q or LysM–TD linker–lipid.

2-9. Qualitative results of antifungal activity test for thrombin

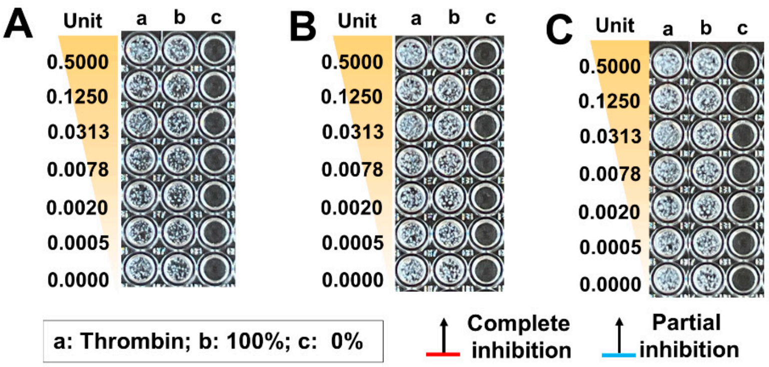

Figure S8. Representative image of a 96-well plate after culturing *C.albicans* in the presence of 0–0.5 Unit of thrombin at (A) 24 h, (B) 48 h and (C) 72 h in 20 mM NaPi, pH 7.4, humidity 90% at 35°C.

2-10. Evaluation of the cleavage of TD linker by thrombin

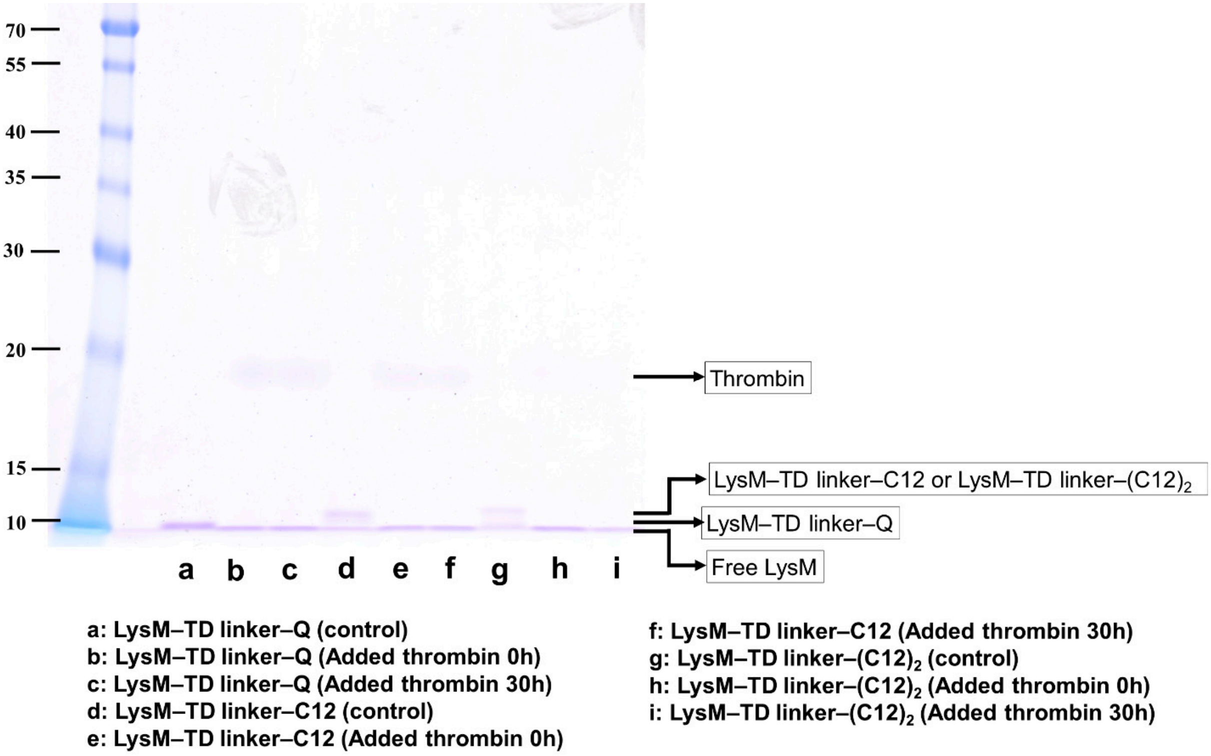

Figure S9. SDS-PAGE analysis of thrombin activity assay after different incubation time of LysM–TD linker–Q, LysM–TD linker–C12 and LysM–TD linker–$(C12)_2$ at 35°C.
